# Purification and Characterization of a Fucoidanase (FNase S) from a Marine Bacterium *Sphingomonas paucimobilis* PF-1

**DOI:** 10.3390/md13074398

**Published:** 2015-07-16

**Authors:** Woo Jung Kim, Joo Woong Park, Jae Kweon Park, Doo Jin Choi, Yong Il Park

**Affiliations:** 1 Department of Biotechnology and The Biomaterial Engineering Research Center, The Catholic University of Korea, Bucheon, Gyeonggi-do 420-743, Korea; E-Mails: wj0504@gstep.re.kr (W.J.K.); pjwnbd@hanmail.net (J.W.P.); myenwls@hanmail.net (D.J.C.); 2 Biocenter, Gyeonggi Institute of Science and Technology Promotion (GSTEP), Suwon, Gyeonggi-do 443-270, Korea; 3 Department of Pharmaceutical Science, University of Gachon, Yeonsu-dong, Yeonsu-gu, InCheon 406-799, Korea; E-Mail: jkpark@gachon.ac.kr

**Keywords:** fucoidan, *Sphingomonas* sp., fucoidanase, galactofuco-oligosaccharides

## Abstract

The Search for enzyme activities that efficiently degrade marine polysaccharides is becoming an increasingly important area for both structural analysis and production of lower-molecular weight oligosaccharides. In this study, an *endo*-acting fucoidanase that degrades Miyeokgui fucoidan (MF), a sulfated galactofucan isolated from the sporophyll (called *Miyeokgui* in Korean) of *Undaria pinnatifida*, into smaller-sized galactofuco-oligosaccharides (1000–4000 Da) was purified from a marine bacterium, *Sphingomonas paucimobilis* PF-1, by ammonium sulfate precipitation, diethylaminoethyl (DEAE)-Sepharose column chromatography, and chromatofocusing. The specific activity of this enzyme was approximately 112-fold higher than that of the crude enzyme, and its molecular weight was approximately 130 kDa (FNase S), as determined by native gel electrophoresis and 130 (S1), 70 (S2) and 60 (S3) kDa by sodium dodecyl sulfate-polyacrylamide gel electrophoresis (SDS-PAGE). The optimum pH and temperature of FNase S were pH 6.0–7.0 and 40–45 °C, respectively. FNase S activity was enhanced by Mn^2+^ and Na^+^ (115.7% and 131.2%), but it was inhibited by Ca^2+^, K^+^, Ba^2+^, Cu^2+^ (96%, 83.7%, 84.3%, and 89.3%, respectively), each at 1 mM. The *K_m_*, *V_max_* and *K_cat_* values of FNase S on MF were 1.7 mM, 0.62 mg·min^−1^, and 0.38·S^−1^, respectively. This enzyme could be a valuable tool for the structural analysis of fucoidans and production of bioactive fuco-oligosaccharides.

## 1. Introduction

Fucoidans are a group of sulfated polysaccharides that exhibit various biological activities including anti-viral, anti-bacterial, anticoagulant and anti-tumoral activities [1,2,3,4]. The biological activities of fucoidans vary depending on the sources. The fucoidans of brown algae represent a family of homo- and hetero-polysaccharides that are mainly composed of fucose residues that are sulfated at positions 2 and/or 4 [5] and linked through α-1,2-, α-1,3-, or α-1,4-*O*-glycosidic bonds [6,7]. Fucoidans have also been demonstrated to contain minor amounts of mannose, xylose, galactose, glucose, uronic acids, and rhamnose [8]. It is generally believed that monosaccharide composition and sulfate contents are related to the different biological activities of fucoidans.

Despite their diverse pharmacologic activities, the structural variation, high molecular masses, and viscous nature of polysaccharides including marine-derived ones may limit their successful applications, especially as therapeutic agents, in terms of problems in polysaccharide standardization, solubility, and bioavailability [9,10]. Bioactive oligosaccharides with lower-molecular weights would help overcome these problems. Indeed, recently, significant efforts have been made to prepare oligosaccharides from marine polysaccharides, aiming at the pharmaceutical usage of bioactive oligosaccharides [8,9,10,11,12]. For example, the fuco-oligosaccharides produced by the hydrolysis of fucoidan with fucoidanases have been applied as pharmaceutical materials in medical sciences [13]. On the other hand, fucoidanases can be either *exo*- or *endo*-acting enzymes. However, no commercial *endo*-fucosidases [5] are currently available.

Therefore, searching for new enzyme activities that degrade marine polysaccharides becomes increasingly important area for both academic researchers and industry people. Especially, finding new fucoidanases and their subsequent kinetic characterizations would provide not only insight into the relationships between the structures and the biological activities of fucoidans but also improved technologies for the production of industrially important bioactive fuco-oligosaccharides [11]. Thus, enzymes having fucoidan-degrading activities have been sought for enzymatic modifications of fucoidans for the preparation of lower-molecular weight fuco-oligosaccharides. The major advantage for the enzymatic digestion over the chemical cleavage is the preservation of sulfate groups on fucoidans, which are recognized as the major factors implicated in the various biological activities of sulfated polysaccharides (SPS).

Previously, we reported on the isolation and identification of a marine bacterial strain, *Sphingomonas paucimobilis* PF-1, that degrades the Miyeokgui fucoidan (MF), which was purified in house from the sporophyll (commonly called *Miyeokgui* in Korean and *Mekabu* in Japanese) of Korean *Undaria pinnatifida* [14]. When intact cells of this strain was used as an enzyme source and incubated with MF as the substrate, seven distinct low-molecular weight fuco-oligosaccharides (LMFOs) with masses lower than approximately 4 kDa, were produced [14]. On the other hand, we also reported that the MF fucoidan is actually an *O*-acetylated sulfated galactofucan polysaccharide with a molecular mass of 1246 kDa, as characterized by various methods, such as organic elemental analysis, high performance liquid chromatography (HPLC) analysis of neutral sugars, Fourier transform infrared spectroscopy (FTIR), FT-Raman (Fourier transform-Raman), ^1^H and ^13^C nuclear magnetic resonance (NMR) [15].

In the present study, we first report on the purification and characterization of a fucoidanase (FNase S) from *Sphingomonas paucimobilis* PF-1, which degrades the high-molecular weight fucoidan (MF) into low-molecular weight galactofuco-oligosaccharides.

## 2. Results and Discussion

### 2.1. Enzyme Purification

**Figure 1:**
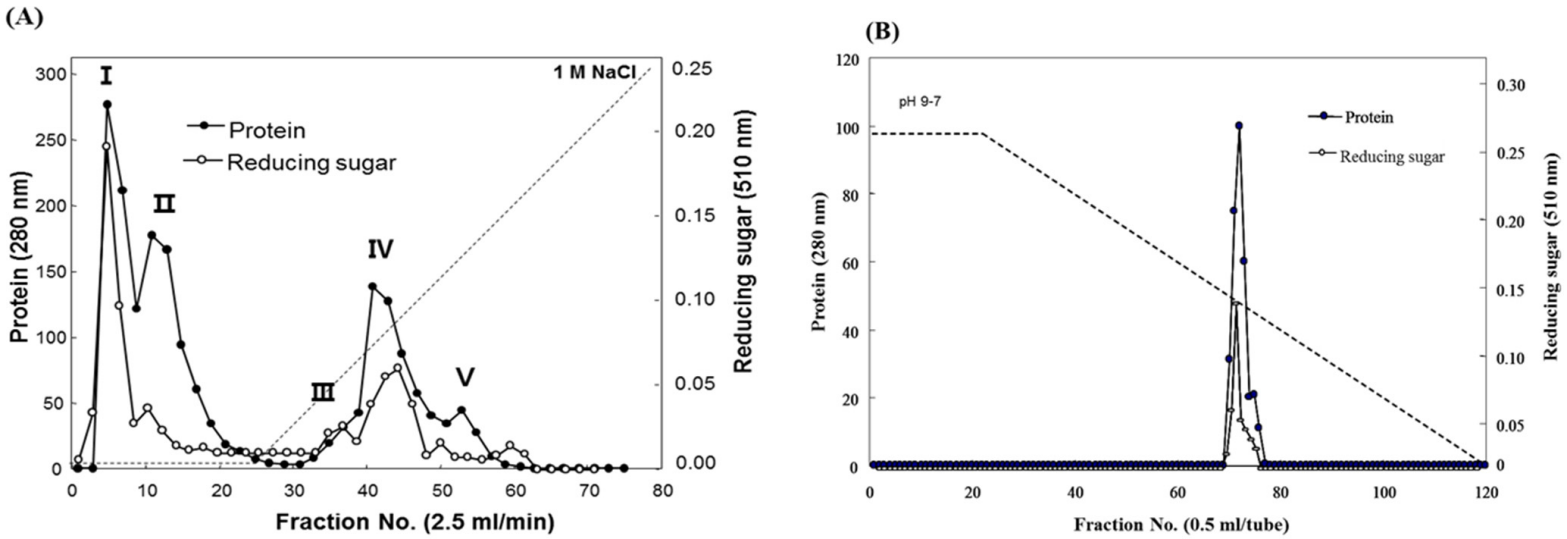
Elution profiles of the fucoidanase from *Sphingomonas paucimobilis* PF-1 resulting from (**A**) diethylaminoethyl (DEAE)-Sepharose fast-flow anion exchange column chromatography and (**B**) chromatofocusing.

Fucodianase was purified from the supernatant that was prepared from the cell harvest via ammonium sulfate fractionation, diethylaminoethyl (DEAE)-Sepharose Fast-Flow anion-exchange chromatography and chromatofocusing. Consequently, the elution profile of the DEAE Sepharose chromatography indicated that among the five peaks with fucoidanase activities, fractions 2 to 8 (peak I) exhibited relatively higher peak than the others (Figure 1A). The fractions in peak I were collected and concentrated with 80% (NH_4_)_2_SO_4_. The elution profile from the chromatofocusing revealed a single peak with fucoidanase activity that appeared from fractions 71 to 75 (Figure 1B). These results, summarized in Table 1, indicate that the enzyme was purified to homogeneity, and a 112.8-fold increase in the specific fucoidanase activity (relative to the activity of the cell disruption supernatant) with a yield of approximately 3.2%. Notably, all of the processes employed in the purification step may provide useful tools for the purification of those displayed on the cell surface since we previously reported that this fucoidan-degrading enzyme activity of *S. paucimobilis* PF-1 is observed with the harvested intact cells, implying its localization on the surface of the cell [14].

**Table 1:** Isolation and purification of fucoidanase from *Sphingomonas paucimobilis* PF-1.

Step	Total volume (mL)	Protein (mg/mL)	Total protein (mg)	Activity (mg/mL)	Specific activity (U·mg^−1^ of protein)	Total activity (unit)	Yield (%)
Cell disruption supernatant	100	44.287	4428.7	0.841	0.019	84.08	100
80% Ammonium sulfate	70	19.582	1370.74	0.538	0.027	37.66	44.79
DEAE-Sepharose	15	0.147	2.205	0.210	1.429	3.15	3.75
Chromatofocusing	3	0.042	0.126	0.090	2.143	0.27	3.2

### 2.2. Estimation of the Molecular Mass and N-Terminal Sequence of the Fucoidanase

**Figure 2:**
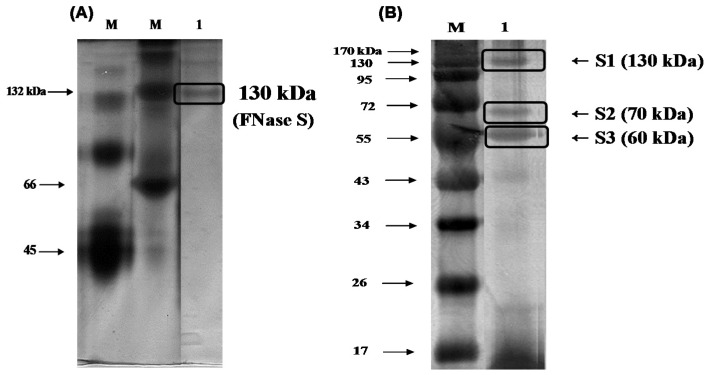
Gel-electrophoresis analyses of the purified enzyme. The purified fucoidanase was analyzed with (**A**) 12% native-polyacrylamide gel electrophoresis (PAGE) and (**B**) sodium dodecyl sulfate (SDS)-PAGE, and stained with silver. M, molecular weight marker (kDa); lane 1, purified fucoidanase on the native-PAGE (**A**); lane 1, purified fucoidanase on the SDS-PAGE performed without mercaptoethanol in the sample buffer (**B**).

To determine the protein purity and molecular mass of the purified enzyme, native-polyacrylamide gel electrophoresis (PAGE) and sodium dodecyl sulfate (SDS)-PAGE was used according to the methods described by Laemmli [16]. The results shown in Figure 2A indicated that there was one single protein band from the final concentrated elutant, and the relative molecular mass of the purified fucoidanase was estimated to be 130 kDa (FNase S) with native-PAGE. It has been reported that the majority of *Flavobacteriaceae* sp. fucoidanases have molecular masses of 105 kDa, based on SDS-PAGE [17]. In general, the high-molecular weight enzyme (e.g., FNase S) can be dissociated by treatment with SDS and boiling for 10 min into several components that can be separated with SDS-PAGE. To determine whether the protein was monomeric, the purified enzyme was analyzed with SDS-PGAE. As shown in Figure 2B, the isolated multi-complex enzyme exhibited three subunits that were derived from the purified enzyme, appeared as a single band in the native-PAGE, and were determined to have molecular weights of 130 (S1), 70 (S2), and 60 kDa (S3) by SDS-PAGE. A similar result was reported by another research group. Furukawa *et al.* [18] reported that the molecular masses of the fucoidanase E1, E2, and E3 of the *Vibrio* sp. N-5 were 39 kDa, 68 kDa, and 68 kDa, respectively, based on SDS-PAGE analysis. Although the evidence is insufficient to reach a clear conclusion, these results indicated that the purified fucoidanases from both of the bacterial strains appeared to be similar to the large extracellular enzyme complex, called the cellulosome, of the plant-cell-wall-degrading anaerobic microorganisms [19]. The cellulosome consists of a scaffolding protein and many bound enzymes. Similarly, the subunits of FNase S of *Sphingomonas paucimobilis* PF-1 are probably involved in the enzyme’s activity, stability and/or binding to substrate in physiological conditions.

The *N*-terminal 10-amino acid sequences of the S1, S2, and S3 subunits were analyzed and determined to be SXPEAASLPG, SPQFDVVXIG, and SLQFDVVVIG, respectively. A BLASTp search of GENBANKnr using the three domains revealed homologies primarily with hypothetical bacterial conserved proteins, with various annotations, such as protein-containing ATPase, core domain (EGF 27867), carbohydrate kinase, PfkB family protein (YP003854323) and dihydrolipoyl dehydrogenase (ZP08017938) (Table 2), which are not related to any glycohydrolases or glycosidases. Based on these results, we suggest that the purified protein might be a unique enzyme with fucoidanase activity, although full amino acid sequences of these subunits need to be further determined in the future.

**Table 2:** Determinations of the *N*-terminal amino acid sequences of (**A**) S1, (**B**) S2, and (**C**) S3. The isolated multi-complex enzyme was composed of three subunits with molecular weights of 130 (S1), 70 (S2), and 60 kDa (S3), as determined with SDS-PAGE.

Matched protein	Mol. Mass (kDa)	Positives (%)	Matched species	Functional category	Matched Peptide	Accession No.
(**A**) The homology comparison of *N*-terminal 10-amino acid sequence of the S1 subunit to the hypothetical bacterial conserved proteins by a BLASTp search.						
Protein containing ATPase, AAA+ type, core domain	244	8/10 (80)	*Rhodopirellula baltica* WH47	Protein containing ATPase, core domain	maed**a**s**peaaslpg**aagdgg	EGF 27867
Hypothetical protein RB6669	202	8/10 (80)	*Rhodopirellula baltica* SH 1	ATP binding site	maedas**peaaslpg**aagdgg	NP 867389
Chitinase	127	8/10 (80)	*Arthroderma otae* CBS 113480		wp**d**v**ldaaslp**tltletagg	XP002842640
ABC sugar (glycerol) transporter, inner membrane subunit	32	8/10 (80)	*Sagittula stellata* E-37	Inner membrane subunit	madtt**s**a**p**g**aaslpg**dvtak	ZP01747232
Glycoside hydrolase family protein	103	8/10 (80)	*Roseiflexus castenholzii* DSM 13941	Sugar binding domain	sls**d**g**pe**s**aslpg**rfplret	YP001433316
(**B**) The homology comparison of *N*-terminal 10-amino acid sequence of the S2 subunit to the hypothetical bacterial conserved proteins by a BLASTp search.						
Carbohydrate kinase, PfkB family protein	34	8/10 (80)	*Parvularcula bermudensis* HTCC2503	Adenosine_kinase	ma**pqfdvi**a**ig**naivdllah	YP003854323
Hypothetical protein DFA_11777	78	9/10 (90)	*Dictyostelium fasciculatum*	Hypothetical protein	mvs**phydvv**v**ig**agiaglsq	EGG14015
Dihydrolipoamide dehydrogenase	50	8/10 (80)	*Xanthomonas axonopodis* pv. *citri str. 306*	Pyridine nucleotide-disulphide oxidoreductase	m**s**e**qfdvv**v**ig**agpagyhaa	NP641866
Sugar kinase	34	8/10 (80)	*Erythrobacter* sp. SD-21	Adenosine kinase	mt**dp**r**ydvv**a**ig**naivdvma	ZP01863524
(**C**) The homology comparison of *N*-terminal 10-amino acid sequence of the S3 subunit to the hypothetical bacterial conserved proteins by a BLASTp search.						
Dihydrolipoyl dehydrogenase	51	9/10 (90)	*Lautropia mirabilis* ATCC 51599	Dihydrolipoyl dehydrogenase	m**slefdvvvig**agpggyiaa	ZP08017938
Anaerobic glycerol-3-phosphate dehydrogenase subunit B	45	9/10 (90)	*Serratia odorifera* DSM 4582	ANAEROBIC glycerol-3-phosphate dehydrogenase	**mqfdvvvig**g	ZP06638221
Transmembrane protein	42	9/10 (90)	*Chromobacterium violaceum* ATCC 12472	Transmembrane protein	m**qfdvivig**a	NP902173

AAA+, ATPases associated with various cellular activities; ABC, ATP-binding cassette.

### 2.3. Optimum pH and Temperature for Enzyme Activity and Stability

**Figure 3:**
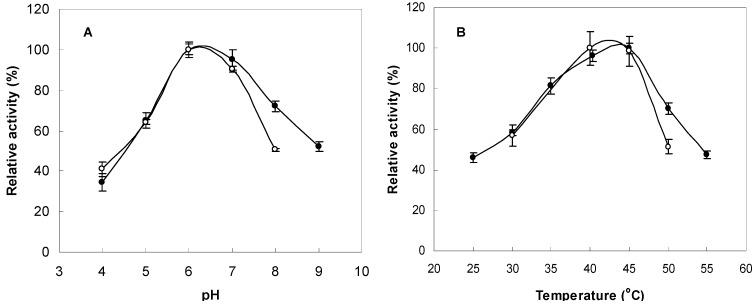
Effects of pH and temperature on enzyme activity and stability. (**A**) The enzyme activities were measured at various pH levels at 45 °C for three days (●). The enzyme solutions were preincubated at various pH levels for one day, and the remaining activities were measured at pH 6.0 and 45 °C for three days (○). (**B**) The enzyme activities were measured at various temperatures in 10 mM sodium acetate buffer at a pH of 6.0 for three days (●). The enzyme solutions were preincubated at various temperatures for one day in 10 mM sodium acetate buffer (pH 6.0), and the remaining activities were measured at 45 °C for three days (○).

The FNase S activity was measured at various pH values (pH 4.0~9.0) in buffers with the same ionic concentrations to determine the optimal conditions. The pH stability was tested via 24-h preincubations of the FNase S in appropriate buffers at 45 °C that had the same ionic concentrations but different pH values that ranged from 4.0 to 9.0. The maximum activity was observed between pH 6.0 and 7.0 (Figure 3A). The FNase S activity was measured as a function of temperature from 25 °C to 55 °C, and the activity was found to be greatest at 45 °C (Figure 3B). The thermostability was tested after preincubation at various temperatures (25–55 °C) for one day at pH 6.0 and then by measuring the residual fucoidanase activity at 45 °C. As shown in Figure 3B, this enzyme was shown to be stable at 40–45 °C. The optimum pH and temperature for this enzyme were very close to the enzyme of the marine *Vibrio* sp., which has optimum pH and temperature ranges of pH 6.0 to 8.0 and 35 to 45 °C, respectively [18].

### 2.4. Effects of Meal Ions and Substrate Specificities on the Enzyme Activities

The effects of metal ions on activity of FNase S were investigated (Table 3A). Mn^2+^ and Na^+^ (concentrations of 1 mM) caused significant increases of 115% and 131%, respectively, while Ca^2+^ (86%), K^+^ (83%), Ba^2+^ (84%) and Cu^2+^ (89%) caused significant decreases in enzyme activity. FNase S may require mono- or divalent metal ions (Mn^2+^ and Na^+^) for effective enzyme activity or digestion of Miyeokgui fucoidan (MF) via the metabolic processes that occur in marine environments. The substrate specificities of FNase S on various carbohydrates were investigated (Table 3B). A commercial fucoidan (FF, Sigma, St. Louis, MO, USA) and alginate stimulated the FNase S enzyme activity by 134% and 129%, respectively, when compared with that (100%) on MF as the control. However, heparin (79%), starch (25%), laminarin (54%) and dextran (15%) acted to decrease the activity of FNase S. Therefore, we concluded that FNase S exhibits substrate specificities for fucoidan and alginate. However, to more clearly define the relationships between the molecular structures of these polysaccharides and the enzyme activities, additional comparison studies with other fucoidanases are needed. Unfortunately, to date, less is known about the enzymatic properties of fucoidanases. It is very important to achieve a detailed understanding of the key factors, such as the sulfur content, the sugar composition and the molecular weight, of substrates involved in the enzymatic characteristics.

**Table 3:** Effects of metal ions (**A**) and substrate specificities (**B**) on fucoidanase activities. The data are given as the means ± SD (standard deviation), *n* = 3. (**A**) Control, the fucoidanase activity in the absence of metal ions was regarded as 100%. (**B**) Control, the activity of the purified enzyme (FNase S) on MF was regarded as 100%.

Metal ions (1 mM)	Relative activity (%)
(**A**) Effects of metal ions on fucoidanase activities.	
Control	100
Na^+^	131.2 ± 0.83
Mn^2+^	115.7 ± 0.93
K^+^	83.7 ± 0.26
Cu^2+^	89.3 ± 2.06
Ca^2+^	86 ± 1.68
Ba^2+^	84.3 ± 2.8
(**B**) Effects of substrate specificities on fucoidanase activities.	
**Substrate**	**Relative activity (%)**
Control	100
FF *	134.5 ± 1.9
Heparin	79.2 ± 1.4
Alginate	129.4 ± 2.3
Starch	25.2 ± 0.8
Laminarin	54.9 ± 2.2
Dextran	15.5 ± 3.1

* FF, a commercial fucoidan (Sigma).

### 2.5. Kinetic Parameters

**Figure 4:**
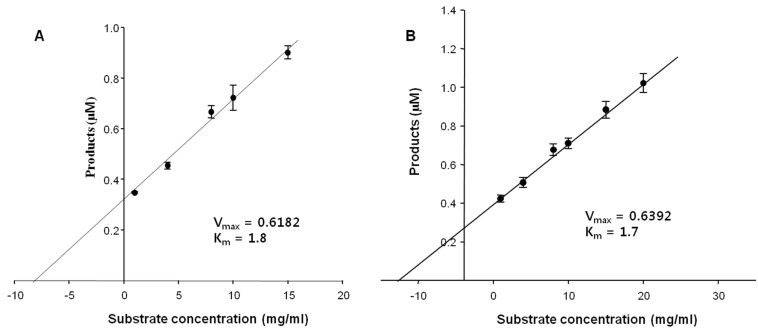
Assay of the enzyme kinetics at different substrate concentrations. The concentration of the enzyme was fixed at 1 mg/mL. (**A**) Miyeokgui fucoidan (MF) and (**B**) FF (commercial fucoidan, Sigma) were used as the substrates. The values of the products were calculated by measuring the reducing sugars released from the substrate fucoidans, (**A**) MF and (**B**) FF, and were expressed in molar concentrations.

Initially, the kinetic parameters of the FNase S against the natural substrates, FF and MF, were determined to be *K*_m_ = 1.7 and 1.8 mM, and *V*_max_ = 0.62 and 0.64 mg/min, respectively (Figure 4). These results also indicated that the *K*_cat_ values of the FNase S were 0.376 S^−1^ and 0.343 S^−1^ (*V*_max_/*K*_m_), respectively (Table 4). Interestingly, MF and FF were hydrolyzed at similarly fast rates (*K*_cat_) and high *K*_cat_/*K*_m_ values. As demonstrated in Figure 3 and Figure 4, the determination of the kinetic parameters and substrate specificities of FNase S are important for understanding its enzymatic properties. However, our ability to find valuable information from other comparable studies was limited due to the paucity of such studies. Therefore, our current date could be the most valuable in terms of its contributions to kinetic studies of the activities of fucoidanases and other related enzymes.

**Table 4:** Kinetic parameters of the purified enzyme (FNase S) on Miyeokgui fucoidan (MF) and commercial fucoidan (FF) as the substrates.

Substrate	*K*_cat_ (S^−1^)	*K*_m_ (mM)	*V*_max_ (mg/min)	*K*_cat_/*K*_m_ (S^−1^·mM^−1^)
FF	0.376 ± 0.04	1.7 ± 0.36	0.62	0.221
MF	0.343 ± 0.1	1.8 ± 0.25	0.64	0.19

FF, commercial fucoidan (Sigma); MF, Miyeokgui fucoidan.

### 2.6. Production and Characterization of Galactofuco-Oligosaccharides by FNase S

**Figure 5:**
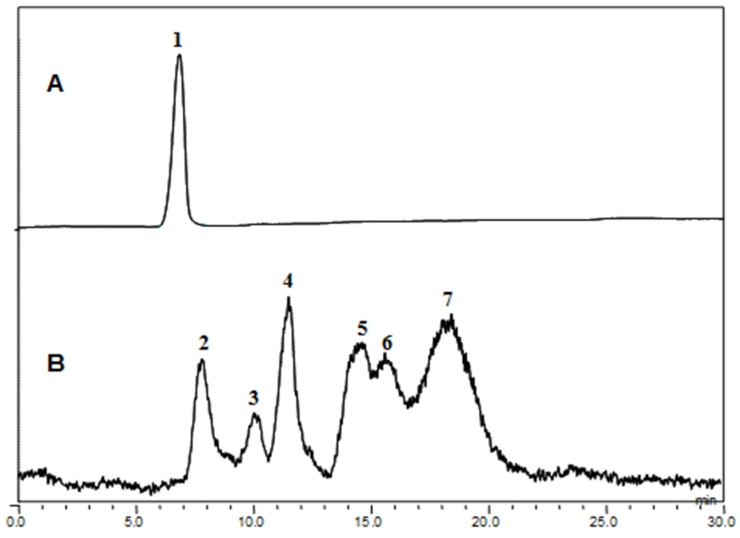
High performance liquid chromatography (HPLC) analysis of Miyeokgui fucoidan (MF) and its enzymatic hydrolysates produced by FNase S. (**A**) MF and (**B**) its hydrolysates were analyzed on a Shodex OHpak SB-806HQ column using HPLC system equipped with evaporative light scattering detector (ELSD). The elution was performed with distilled water at a rate of 0.8 mL/min.

**Figure 6:**
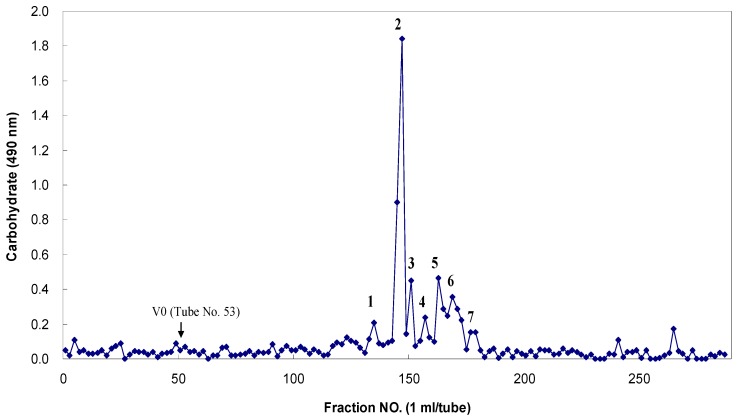
Bio-Gel P-4 column chromatography of the fragmented Miyeokgui fucoidan (MF) after digestion by FNase S.

The fucoidanase activity was determined by measuring the level of reducing sugars and confirmed by HPLC analyses of the degradation products (Figure 5). The fucoidan-degrading activity was also clearly confirmed by the fractionation of the degradation products through Bio-Gel P-4 size-exclusion chromatography and the analysis of the monosaccharide compositions of the resulting oligosaccharides (Figure 6). The oligosaccharides generated were resolved into seven distinct low-molecular mass fractions with peaks that ranged from 1 to 7. Based on comparisons with the masses of the standard malto-oligosaccharides (1–7 glucose units, Sigma), the relative molecular masses of these fucoidan oligosaccharides were determined to be 3312 Da (peak 1), 2494 Da (peak 2), 1699 Da (peak 3), 1543 Da (peak 4), 1312 Da (peak 5), 817 Da (peak 6), and 318 Da (peak 7) (Table 5). Based on the total sugar levels, one major fraction (peak 2) represented approximately 72% of the initial oligosaccharide mixture. Based on the high performance anion-exchange chromatography with pulsed amperometric detection (HPAEC/PAD) analysis that followed trifluoroacetic acid (TFA) hydrolysis, the constituent monosaccharides of these seven major oligosaccharides were determined to be the following: peak 1, fucose, galactose, mannose and xylose at 8:7:2:2 (given as the mole ratios); peak 2, 3:8:2:2; peak 3, 5:3:1:1; peak 4, 3:3:2:1; peak 5, fucose and galactose at 7:1; peak 6, fucose and galactose, 4:1; peak 7, fucose only (dimer) (Table 5). The uronic acid and sulfate contents of these fractions were not determined at this time. These results clearly indicated that fucose and galactose were the major monosaccharides in these oligosaccharides that were derived from MF fucoidan.

**Table 5:** Monosaccharide compositions and relative molecular masses of the galactofuco-oligosaccharides generated from MF by FNase S.

Peak No ^a^	M_r_^b^ (Da)	Monosaccharide composition ^c^	Relative amount (%) ^d^
1	3312	Fucose, Galactose, Mannose, Xylose (mole ratio, 8:7:2:2)	4.5
2	2494	Fucose, Galactose, Mannose, Xylose (mole ratio, 3:8:2:2)	72
3	1699	Fucose, Galactose, Mannose, Xylose (mole ratio, 5:3:1:1)	6.5
4	1543	Fucose, Galactose, Mannose, Xylose (mole ratio, 3:3:2:1)	2.7
5	1312	Fucose, Galactose (mole ratio, 7:1)	5.3
6	817	Fucose, Galactose (mole ratio, 4:1)	6.1
7	318	Fucose (2 moles)	2.9

^a^ The oligosaccharides were fractionated through a Bio-Gel P-4 column after digestion of MF by FNase S; ^b^ The relative molecular mass of each oligosaccharide was estimated using malto-oligosaccharides (1–7 glucose units, Sigma) as standard molecular markers; ^c^ The monosaccharide compositions were determined with high performance anion-exchange chromatography with pulsed amperometric detection (HPAEC-PAD) analyses; ^d^ The carbohydrate contents of each peak were the total neutral sugars as determined with the phenol-sulfuric acid method and the relative amounts of each oligosaccharide were estimated by setting the sum of the major oligosaccharides (peak 1–7) to 100%.

This fucoidanase activity of FNase S was not related to α-l-fucosidase. While the commercial α-l-fucosidase released *p*-nitrophenol from the artificial substrate, *p*-nitrophenyl-α-l-fucoside, our fucoidanase enzyme preparation (FNase S) did not produce *p*-nitrophenol, which demonstrated that the fucoidanase activity of this enzyme in the degradation of MF was not related to the α-l-fucosidase activity (data not shown). Taken collectively, these results strongly suggested that FNase S produces low-molecular weight galactofuco-oligosaccharides from MF, a sulfated galactofucan of the sporophyll (called *Miyeokgui* in Korean) of Korean *Undaria pinnatifida*, and that this FNase S is not an α-l-fucosidase but is rather an *endo*-acting fucoidanase that randomly attacks the fucoidan chains as other reports have demonstrated [8,20].

Bakunina [21] reported on the 25 strains of epiphytic marine bacteria isolated from the brown algae *Fucus evanescens* and *Chorda filum*, and 53 bacterial strains isolated from the sea cucumber *Apostichopus japonicus* were screened for having fucoidanase activity using fucoidans prepared from the brown algae *F. evanescens* and *Laminaria cichorioides* (Table 6)*.* The bacterial epiphytes *Cytophaga* sp. and some bacterial isolates of the genera *Alteromonas* and *Pseudoalteromonas* from the sea cucumber were also found to possess fucoidanase activity [13]. However, the enzyme activity was comparable but lower than that of the already known microbial fucoidanases [22,23]. Although very little is known about the genes that encode fucoidanases, the molecular cloning and biochemical characterization of a special fucanase FcnA termed fucan α-1,4-endohydrolase isolated from the family *Flavobacteriaceae* have decoded the structural basis of sulfated polysaccharides [17,24]. The results of this study demonstrated that our FNase S produces low-molecular weight galactofuco-oligosaccharides from fucoidan (actually a sulfated galactofucan). However, these results raise the questions on how bacteria that produce fucoidanase can take up dimer and larger oligosaccharides into the cytoplasmic space through the cell wall for use as nutrients. Additional studies of the catabolic cascade of MF and its oligomers are underway. In addition, mining and molecular cloning of corresponding genes for FNase S in the future would stimulate the applicability of this enzyme to the various industries for the development of new nutraceuticals, cosmeceuticals, as well as pharmaceuticals.

**Table 6:** Fucoidan-degrading enzymes and their properties.

Producing microorganism	MW (Da)	Habitat	Substrate	Cleaving pattern	Refs.
*Cytophaga*, *Alteromonas*, *Pseudoalteromonas*	n.d.	Intra-cellular	Fucoidan from *Fucus evanescens*, *Laminaria cichorioides* and *Laminaria japonica*	Exo	[21]
*Dendryphiella arenaria*	n.d.	Intra-cellular	Fucoidan from *Fucus vesiculosus* and *Laminaria digitata*	n.d.	[25]
*Flavobacteriaceae*	n.d.	Extra-cellular	Fucoidan from *Pevetia canaliculata*	Endo	[24]
*Flavobacteriaceae*	67,000	Extra-cellular	Fucoidan from *Kjellmaniella crassifolia*	Endo	[26]
*Littorina kurila*	n.d.	Intra-cellular	Fucoidan from *Fucus distichus*	Disaccharides	[27]
*Pseudoalteromonas atlantica*	n.d.	Extra-cellular	Fucoidan from brown seaweed	Exo	[28]
*Patinopecten yessoensis*	85,000	Intra-cellular	Fucoidan from *Nemacystus decipieus*	Endo	[20]
*Pecten maximus*	200,000	Extra-cellular	Fucoidan from *Ascophyllum nodosum,*	endo	[29]
*Pecten maximus*	n.d.	Intra-cellular	Fucoidan from *Ascophyllum nodosum*	Releases l-fucose	[30]
*Pecten maximus*	n.d.	Intra-cellular	Sulfated l-fucopyranoside	Sulfoesterase	[31]
*Vibrio* sp. N5	40,000–68,000	Extra-cellular	Fucoidan from *Kjellmaniella crassifolia*	Endo	[18]
*Fucophilus fucidanolyticus*	n.d.	Intra-cellular	Fucoidan from *Cladosiphon okamuranus*	Endo	[26]
Gram-negative bacterium	67,000	Extra-cellular	Fucoidan from *Kjellmaniella crassifolia*	Endo	[32]
*Sphingomonas paucimobilis PF-1*	≥132,000	Extra-cellular	Fucoidan *from Undaria pinnatifida* sporophyll	Endo	This study

n.d., not determined.

FNase S was incubated with 1% fucoidan for three days at 45 °C in 50 mM sodium acetate buffer (pH 5.6) containing 0.01% sodium azide. The reaction products were eluted with 50 mM sodium nitrate at a flow rate of 0.5 mL/min. Each fraction was monitored for carbohydrates with the phenol-H_2_SO_4_ method at 490 nm using l-fucose as a reference. The arrow indicates the void volume at which the unhydrolyzed fucoidan and hydrolyzed products with larger molecular masses (over approximately 5000 Da) reached the upper exclusion limit of the gels. Only major peaks (1 to 7) were collected and examined for their monosaccharide compositions with high performance anion-exchange chromatography with pulsed amperometric detection (HPAEC-PAD) analyses.

## 3. Experimental Section

### 3.1. Preparation of Fucoidan

Miyeokgui fucoidan (MF) was extracted and purified as described previously [33]. Briefly, fucoidan was extracted from the dried sporophylls (called *Miyeokgui* in Korean) of *Undaria pinnatifida* (called *Miyeok* in Korean and *Wakame* in Japanese) that were collected from the southern coastal area of Wando, Korea, mainly through 0.1 N HCl extraction and 75% ethanol precipitation. The crude fucoidan obtained was further purified through DEAE-Cellulose column chromatography. Commercial fucoidan (tentatively named FF in this study) from alga *Fucus vesiculosus* was purchased from Sigma Chemical (St. Louis, MO, USA).

### 3.2. Microorganism and Culture Conditions

The growth of *Sphingomonas paucimobilis* PF-1 (KCTC 11130BP) was performed as previously reported by our group [14]. The cells were cultured in a minimal medium consisting of 0.5% MF with 2% Bacto peptone in dH_2_O (pH 7.0) at 30 °C for 4 days on a shaking incubator (180 rpm, JEIO TECH Co., Seoul, Korea) and then centrifuged at 6000 rpm, 4 °C for 30 min. The cells harvested were stored at −20 °C and used for the purification of the fucoidanolytic enzymes.

### 3.3. Production and Purification of Fucoidanase

All experiments were performed at 4 °C unless otherwise stated. The cells (50 g, wet weight) were homogenized on ice using a Sonifier 450 (Branson, Danbury, CT, USA) for 1 h in 50 mL of 50 mM sodium acetate buffer (pH 5.6). The homogenate was centrifuged at 16,000 rpm for 40 min to remove the insoluble materials. The supernatant solution was gradually brought up to 40% (w/v) saturation with (NH_4_)_2_SO_4_ and then centrifuged at 16,000 rpm for 30 min to remove the precipitates. Continuously, the supernatant solution was further brought to 80% (w/v) saturation with (NH_4_)_2_SO_4_ and precipitated overnight at 4 °C. The precipitate was collected by centrifugation at 16,000 rpm for 30 min, dissolved in 10 mL of 50 mM sodium acetate buffer (pH 5.6), and dialyzed (10 kDa molecular weight cut off) extensively against 2 L of the same buffer for 3 days at 4 °C and stored at −20 °C. The dialyzed solution (20 mL) was loaded onto a column (2.8 × 30 cm) of DEAE-Sepharose (GE Healthcare, Marlborough, MA, USA) and equilibrated with 10 mM Tris-HCl buffer containing 10 mM sodium chloride (pH 7.2). The column was washed with the same buffer and then was eluted with a linear gradient of 0–1 M sodium chloride. The non-binding fractions on the DEAE-Sepharose were collected and dialyzed against 2 L 0.025 M ethanolamine buffer (pH 9.4). The concentration was applied onto a Mono P (GE Healthcare) column equilibrated with 0.025 M ethanolamine buffer (pH 9.6) and then eluted with a linear gradient of Polybuffer 96 buffer (pH 7.0) (GE Healthcare). The apparent molecular weight of the purified enzyme was estimated with native PAGE and SDS-PAGE analyses using a 12% polyacrylamide gel and Coomassie Brilliant Blue R-250 staining [34]. The molecular masses of the protein standard ranged from 10 to 170 kDa and were prestained on a pro-stain Ladder (Fermentas, Hanover, MD, USA).

### 3.4. Determination of the N-terminal Amino Acid Sequence

The *N*-terminal amino acid residues of fucoidanase were determined using an Applied Biosystems 476A automatic protein sequencer (Amino Acid Sequencing Facility, Institute of Korea Basic Science, Daejeon, Korea). The amino acid sequence was compared with those available in SWISS-PROT and/or the Protein Data Bank.

### 3.5. Effects of pH and Temperature on Fucoidanase Activity and Stability

The optimal temperature for fucoidanase activity was determined over the range of 25 to 55 °C in 50 mM sodium phosphate buffer (pH 6.0) containing 0.5% of MF as the substrate. The thermal stability of the purified enzyme was tested by pre-incubating the enzyme at different temperatures (25 to 55 °C) for 1 day. The remaining activities of fucoidanase were measured immediately after this treatment with the standard method mentioned previously. The activity of fucoidanase was determined by measuring the amount of reducing sugars released from the fucoidan with the Somogyi-Nelson method, using fucose as the standard [35]. One unit of fucoidanase activity was defined as the amount of enzyme (mg) that released 1 nmol of reducing sugar per min. To determine the optimal pH, the fucoidanase activity of the enzyme was studied at 45 °C in the following buffers: 50 mM sodium acetate buffer (pH 4.0 and 5.0), 50 mM sodium phosphate buffer (pH 6.0), and 50 mM Tris-HCl (pH 7.0, 8.0, 9.0). The pH stability of the enzyme was measured based on the residual activity after pre-incubating the enzyme in the above buffer (pH 4.0–9.0) at 45 °C for 1 day.

### 3.6. Effects of Metal Ions on Fucoidanase Activity

The effects of several metal ions on fucoidanase activity were examined in reaction mixtures supplemented with NaCl (Na^+^), MnSO_4_ (Mn^2+^), KCl (K^+^), CuSO_4_ (Cu^2+^), CaCl (Ca^2+^), and BaCl_2_ (Ba^2+^) at final concentrations of 1 mM. The reactions were performed at the optimal pH and temperature over 72 h with MF (0.5%) as the substrate. The fucoidanase activity was determined with the Somogyi-Nelson method as described above.

### 3.7. Determination of the Substrate Specificity

The substrate specificity of the purified enzyme was determined using the following natural substrates: commercial fucoidan (FF) from the alga *Fucus vesiculosus* (Sigma), heparin, alginate, starch, laminarin, and dextran. The purified enzyme (10 μg) was added to 1 mL of 50 mM sodium phosphate buffer (pH 6.0) containing 0.5% substrates. The reactions were performed at the optimal pH and temperature over 72 h. The fucoidanase activity was quantitatively determined by measuring the amount of reducing sugar released from the polysaccharide substrates with the Somogyi-Nelson method, using fucose or glucose as the standard [35].

### 3.8. Determination of the Kinetic Parameters

The hydrolysis kinetics of the purified enzyme on the intact fucoidan (MF) and commercial fucoidan (FF, Sigma) were determined at pH 6.0 and 45 °C with substrate concentrations that varied from 0 to 20 mM and/or 0 to 30 mM, respectively; this step was followed by the standard enzyme assay described above. The *K*_m_, *V*_max_ and *K*_cat_ values were obtained based on a Lineweaver-Burk plot and were expressed as the means of the three different experiments. This plot is widely used to determine important terms in enzyme kinetics, such as *K*_m_ and *V*_max_ for determining the type of enzyme inhibition by using competitive, non-competitive and uncompetitive inhibitors. In this plot, competitive inhibitors have the same *y*-intercept as uninhibited enzyme, since *V*_max_ is unaffected by competitive inhibitors. However, different slopes and x-intercepts between the data sets can be seen in the same plot. On the other hand, non-competitive inhibition has plots with the same *x*-intercept with different slopes and *y*-intercepts. Subsequently, uncompetitive inhibition can cause different intercepts on both the *y*- and *x*-axes. Herewith, our data simply showed in the case of uncompetitive inhibition, since no inhibitor was used to determine the kinetics of fucoidanase. These assays were set up in triplicates. If the plot of 1/V and 1/(substrate) is linear, then the slope is equal to 1/*V*_max_, 1/*K*_m_, and *K*_cat_/*K*_m_.

### 3.9. Size-Exclusion HPLC Analysis for Fucoidanase Activity

Fucoidanase activity was determined by measuring the level of reducing sugars and confirmed with size-exclusion HPLC (Dionex Co., Sunnyvale, CA, USA) using a Shodex OHpak column (SB-806HQ, 8.0 × 300 mm, Showa Denko Co., Tokyo, Japan) under the established conditions, following 75% ethanol precipitation. Next, the supernatant was vacuum-dried using a Speed-Vac (Module spin 40, Biotron, Seoul, Korea), 100 μL dH_2_O was added to dissolve the pellet. Ten microliters of the sample was injected, eluted with water at a flow rate of 0.8 mL/min at 60 °C and detected with an evaporative light scattering detector (ELSD, Deerfield, IL, USA).

### 3.10. Identification of the Galactofuco-Oligosaccharides

The reaction mixture of MF and purified enzyme was fractionated through a Bio-Gel P-4 column (1.5 × 95 cm), and each oligomer eluted from the column was analyzed for its monosaccharide composition and molecular mass. The reaction mixture of 10 mL of 50 mM sodium phosphate buffer (pH 6.0) containing 1% fucoidan and 10 mL of purified enzyme (10 μg/min) was incubated for 3 days at 45 °C in a shaking water bath (120 rpm). After incubation, the enzyme was removed by centrifugation and the supernatant was concentrated by freeze drying. The dried material was dissolved in 5 mL of dH_2_O and precipitated with 75% ethanol. Next, the supernatant was vacuum-dried using a Speed-Vac. The dried material was dissolved in 1 mL of 50 mM sodium nitrate and 0.5 mL of the material was applied to the Bio-Gel P-4 column and eluted at 0.5 mL/min with the same solvent. The fractions containing carbohydrates were pooled together and freeze-dried to determine the monosaccharide compositions of the galactofuco-oligosaccharides. Pooled fractions were dissolved in 0.1 mL of dH_2_O, an equal volume of 4 M trifluoroacetic acid (TFA) was added, and the mixtures were allowed to stand for 4 h at 100 °C with gentle stirring. After the reaction, the mixture was filtered through a 0.45-μm syringe filter and vacuum-dried using a Speed-Vac. The dried material was re-dissolved in 0.1 mL of dH_2_O. The monosaccharide compositions of the TFA-hydrolyzed galactofuco-oligosaccharides were assessed with HPAEC-PAD (Dionex Co.) as described by Lee *et al.* [36].

### 3.11. Statistical Analysis

The statistical significances of the differences between the mean values were determined with Student’s *t*-tests. The experimental values represent the means ± the standard deviations.

## 4. Conclusions

In this study, we purified an enzyme (FNase S) that degrades Miyeokgui fucoidan (MF), which is a sulfated galactofucan, into smaller-sized galactofuco-oligosaccharides (1000–4000 Da), from a strain of *Sphingomonas paucimobilis.* Experiments involving the over-production and the purification of fucoidanases are thus of great interest, as is the processing of these enzymes to make them usable in industrial processes. The naturally occurring fucoidans require further structural elucidation to create a source of pharmaceutical drugs or any value-added raw materials for industrial processes. Additional knowledge about fucoidan-degrading enzymes will facilitate studies that aim to elucidate the structures of fucoidans and other fucoidan type polysaccharides and enable the production of lower-molecular weight functional galactofuco-oligosaccharides.

## References

[1] Hoshino T., Hayashi T., Hayashi J., Lee J.B., Sankawa U. (1998). An antivirally active sulfated polysaccharide from *Sargassum horneri* (TURNER) C. AGARDH. Biol. Pharm. Bull..

[2] Cumashi A., Ushakova N.A., Preobrazhenskaya M.E., D’Incecco A., Piccoli A., Totani L., Tinari N., Morozevich G.E., Berman A.E., Bilan M.I. (2007). A comparative study of the anti-inflammatory, anticoagulant, anti-angiogenic and anti-adhesive activities of nine different fucoidans from brown seaweeds. Glycobiology.

[3] Alekseyenko T.V., Zhanayeva S.Y., Venediktova A.A., Zvyagintseva T.N., Kuznetsova T.A., Besednova N.N., Korolenko T.A. (2007). Antitumor and antimetastatic activity of fucoidan, a sulfated polysaccharide isolated from the Okhotsk sea *Fucus evanescens* brown alga. Bull. Exp. Biol. Med..

[4] Hahnenberger R., Jakobson A.M. (1991). Antiangiogenic effect of sulfated glycosaminoglycans and polysaccharides in the chick embryo chorioallantoic membrane. Glycoconj. J..

[5] Chevolot L., Foucault A., Chaubet F., Kervarec N., Sinquin C., Fisher A.M., Boisson-Vidal C. (1999). Further data on the structure of brown seaweed fucans: Relationships with anticoagulant activity. Carbohydr. Res..

[6] Usov A.I., Smirnova G.P., Bilan M.I., Shashkov L.S. (1998). Polysaccharides of brown algae. Bioorg. Khim..

[7] Mulloy B., Ribeiro A.C., Alves A.P., Vieira R.P., Mourão P.A.S. (1994). Sulfated fucans from echinoderms have a regular tetrasaccharide repeating unit defined by specific patterns of sulfation at the *O*-2 and *O*-4 positions. J. Biol. Chem..

[8] Berteau O., Mulloy B. (2003). Sulfated fucans, fresh perspectives: Structures, functions, and biological properties of sulfated fucans and an overview of enzymes active toward this class of polysaccharides. Glycobiology.

[9] Silchenko A.S., Kusaykin M.I., Kurilenko V.V., Zakharenko A.M., Isakov V.V., Zaporozhets T.S., Gazha A.K., Zvyagintseva T.N. (2013). Hydrolysis of Fucoidan by Fucoidanase Isolated from the Marine Bacterium, Formosa algae. Mar. Drugs.

[10] Ji J., Wang L.C., Wu H., Luan H.M. (2011). Bio-function Summary of Marine Oligosaccharides. Int. J. Biol..

[11] Holtkamp A.D., Kelly S., Ulber R., Lang S. (2009). Fucoidans and fucoidanases-focus on thchniques for molecular structure elucidation and modification of marine polysaccharides. Appl. Microbiol. Biotechnol..

[12] Courtois J. (2009). Oligosaccharides from land plants and algae: Production and applications in therapeutics and biotechnology. Curr. Opin. Microbiol..

[13] Bakunina I., Nedashkovskaya O.I., Alekseeva S.A., Ivanova E.P., Romanenko L.A., Gorshkova N.M., Isakov V.V., Zvyagintseva T.N., Mikhailov V.V. (2002). Degradation of fucoidan by the marine proteobacterium *Pseudoalteromonas citrea*. Mikrobiologiia.

[14] Kim W.J., Kim S.M., Lee Y.H., Kim H.G., Kim H.K., Moon S.H., Suh H.H., Jang K.H., Park Y.I. (2008). Isolation and characterization of marine bacterial strain degrading fucoidan from Korean *Undaria pinnatifida* sporophylls. J. Microbiol. Biotechnol..

[15] Synytsya A., Kim W.J., Kim S.M., Pohl R., Synytsya A., Kvasnicˇka F., Čopíková J., Park Y.I. (2010). Structure and antitumour activity of fucoidan isolated from sporophyll of Korean brown seaweed *Undaria pinnatifida*. Carbohydr. Polym..

[16] Laemmli U.K. (1970). Cleavage of structural proteins during assembly of head of bacteriophage T4. Nature.

[17] Colin S., Deniaud E., Jam M., Descamps V., Chevolot Y., Kervarec N., Yvin J.C.., Barbeyron T., Michel G., Kloareg B. (2006). Cloning and biochemical characterization of the fucanase FcnA: Definition of a novel glycoside hydrolase family specific for sulfated fucans. Glycobiology.

[18] Furukawa S., Fujikawa T., Koga D., Ide A. (1992). Purification and some properties of *exo*-type fucoidanases from *Vibrio* sp. N-5. Biosci. Biotechnol. Biochem..

[19] Doi R.H., Kosugi A. (2004). Plant-cell-wall-degrading enzyme complexes. Nat. Rev..

[20] Kitamura K., Matsuo M., Yasui T. (1992). Enzymatic degradation of fucoidan by fucoidanase from the hepatopancreas of *Patinopecten yessoensis*. Biosci. Biotechnol. Biochem..

[21] Bakunina I.Yu., Shevchenko L.S., Nedashkovskaya O.I., Shevchenko N.M., Alekseeva S.A., Mikhailov V.V., Zvyagintseva T.N. (2000). Screening of marine bacteria for fucoidanases. Microbiology.

[22] Sakai T., Kimura H., Kojima K., Ikai K., Akiyoshi S., Nakanishi Y., Kato I. (1997). Oligosaccharides manufacture by hydrolysis of fucoidan. Chem. Abstr..

[23] Gonzalez J.M., Weiner R.M. (2000). Phylogenetic characterization of marine bacterium strain 2-40, a degrader of complex polysaccharides. Int. J. Syst. Evol. Microbiol..

[24] Descamps V., Colin S., Lahaye M., Jam M., Richard C., Potin P., Barbeyron T., Yvin J.C., Kloareg B. (2005). Isolation and culture of a marine bacterium degrading the sulfated fucans from marine brown algea. Mar. Biotechnol..

[25] Kelly S., Holtkamp A., Poth S., Lang S., Ulber R. (2008). Untersuchungen zur potenziellen fucoidanase-Aktivität *von Dendryphiella Arenaria*. Chem. Ing. Tech..

[26] Sakai T., Ishizuka K., Shimanaka K., Ikai K., Kato I. (2003). Structures of oligosaccharides derived from *Cladosiphon okamuranus* fucoidan by digestion with marine bacterial enzymes. Mar. Biotechnol..

[27] Bilan M.I., Kusaykin M.I., Grachev A.A., Tsvetkova E.A., Zvyagintseva T.N., Nifantiev N.E., Usov A.I. (2005). Effect of enzyme preparation from the marine mollusk *Littorina kurila* on fucoidan from the brown alga *Fucus distichus*. Biochemistry (Mosc.).

[28] Yaphe W., Morgan K. (1959). Enzymic hydrolysis of fucoidin by *Pseudomonas atlantica* and *Pseudomonas carrageenovora*. Nature.

[29] Berteau O., McCort I., Goasdoue N., Tissot B., Daniel R. (2002). Characterization of a new alpha-l-fucosidase isolated from the marine mollusk *Pecten maximus* that catalyzes the hydrolysis of alpha-l-fucose from algal fucoidan (*Ascophyllum nodosum*). Glycobiology.

[30] Daniel R., Berteau O., Jozefonvicz J., Goasdoue N. (1999). Degradation of algal (*Ascophyllum nodosum*) fucoidan by an enzymatic activity contained in digestive glands of the marine mollusk *Pecten maximus*. Carbohyd. Res..

[31] Daniel R., Berteau O., Chevolot L., Varenne A., Gareil P., Goasdoue N. (2001). Regioselective desulfation of sulfated l-fucopyranoside by a new sulfoesterase from the marine mollusk *Pecten maximus*: Application to the structural study of algal fucoidan (*Ascophyllum nodosum*). Euro. J. Biochem..

[32] Sakai T., Kawai T., Kato I. (2004). Isolation and characterization of a fucoidan-degrading marine bacterial strain and its fucoidanase. Mar. Biotechnol..

[33] Kim W.J., Kim S.M., Kim H.G., Oh H.R., Lee K.B., Lee Y.K., Park Y.I. (2007). Purification and anticoagulant activity of a fucoidan from Korean *Undaria pinnatifida* sporophyll. Algae.

[34] George V., Diwan A.M. (1983). Simultaneous staining of proteins during polyacrylamide gel electrophoresis in acidic gels by countermigration of Coomassie brilliant blue R-250. Anal. Biochem..

[35] Somogyi M. (1952). Notes on sugar determination. J. Biol. Chem..

[36] Lee Y.K., Lim D.J., Lee Y.H., Park Y.I. (2006). Variation in fucoidan contents and monosaccharide compositions of Korean *Undaria pinnatifida* (Harvey) suringar (Phaeophyta). Algae.

